# Outcomes Following Staged Bilateral Total Knee Replacements: The Influence of First-Side Surgery on Results of the Second Side

**DOI:** 10.7759/cureus.70374

**Published:** 2024-09-28

**Authors:** Joseph Boktor, Ullas Jayaraju, Vinay Joseph, Rishi Trivedi, Awf A Alshahwani, Kunal Roy, Peter Lewis

**Affiliations:** 1 Trauma and Orthopaedics, Cardiff University Hospital, Cardiff, GBR; 2 Trauma and Orthopaedics, Royal Glamorgan Hospital, Llantrisant, GBR; 3 Trauma and Orthopaedics, Prince Charles Hospital, Merthyr Tydfil, GBR; 4 Trauma and Orthopaedics, Glangwili General Hospital, Carmarthen, GBR; 5 Trauma and Orthopaedics, Leicester Royal Infirmary, Leicester, GBR; 6 Trauma and Orthopaedics, Leicester University Hospital, Leicester, GBR; 7 Trauma and Orthopaedics, University Hospital of Wales, Cardiff, GBR; 8 Trauma and Orthopaedics, Cwm Taf University Health Board, Merthyr Tydfil, GBR

**Keywords:** bilateral total knee arthroplasty, minimal clinically important difference, oxford knee scores (oks), patient-reported outcome measure, staged total knee arthroplasty

## Abstract

Background

In the UK, the vast majority of bilateral total knee replacements (BTKRs) are performed as staged procedures. Historically, the second-operated knee, in patients undergoing staged BTKRs, has poorer outcomes compared with the first-operated knee. The aim was to review patient-reported outcome measures (PROMs) for staged BTKRs. To further assess if outcomes from the first side of surgery can predict results on the second side.

Methods

A retrospective analysis was conducted on a consecutive cohort of staged BTKRs using the same approach and technique from August 2009 to January 2020. PROMs were recorded by means of Oxford Knee Scores (OKS) preoperatively, at six weeks postoperatively, and at one year. The minimal important change (MIC) in PROMs was set at ≥5.

Results

A total of 186 consecutive staged BTKRs were carried out in 93 patients. The average interval between surgeries was 28 months, ranging from 3 to 100 months. The mean age was 65.3 years for the first side and 67.7 years for the second side, with 58.1% of the patients (54/93) being female. The mean body mass index (BMI) was 35.1 for the first side and increased to 35.8 for the second side (p=0.503). One-year follow-up PROMs were available for 97.8% of the first-side surgeries and 89.2% of the second-side surgeries. The average improvement in PROMs at one year was 18.3 for the first side and 15.8 for the second side (p=0.108). A total of 95.6% of patients achieved the MIC for the first side, while 87.9% achieved it for the second side (p=0.340). Fourteen TKRs performed in 13 patients did not achieve MIC at one year. Among these 13 patients, three (23.1%) did not reach MIC after the first-side surgery, compared to nine (61.5%) after the second-side surgery. One patient did not achieve MIC for either side. Of the 14 TKRs that failed to reach MIC in one year, 10 eventually met MIC in two years. The four TKRs that did not achieve MIC at two years were performed on females with high BMIs.

Conclusion

This study found no significant difference in PROMs improvements between the first and second sides of staged BTKRs at one-year follow-up. A failure to reach the MIC on one side does not affect the success of the other side. Most patients who do not achieve MIC in one year are likely to reach it in two years. Persistent poor scores were associated with high BMI and female sex.

## Introduction

Knee joint replacements are among the most in-demand of all elective surgical operations performed within the National Health Service (NHS) [1,2]. There is a growing need for staged bilateral total knee replacement (BTKR) surgery due to an ever-ageing population who present with bilateral degenerative joint disease [3]. In the United Kingdom (UK), 98.7% of BTKRs are performed as staged procedures [4].

Literature suggests that the second-operated knee, in patients undergoing staged BTKRs, has inferior outcomes compared with the first-operated knee [5]. Furthermore, complications occurring after the first-operated knee were associated with a significantly increased probability of recurrence following the second-side surgery [6].

Patient-reported outcome measures (PROMs) are essential for guiding surgical decisions and enhancing surgical outcomes. According to the NHS England PROMs 2021-2022 report, 95% of total knee replacements (TKRs) showed improvement based on Oxford Knee Scores (OKS), with an average score increase of 17.3 points [7]. Additionally, 90% of knee replacement patients felt better after their surgery, and 87% rated their operative results as excellent, very good, or good [7]. However, these national improvements in OKS scores do not account for the minimal important change (MIC) [8]. MIC represents a threshold for change that patients perceive as significant, beyond statistical variation, and is considered clinically relevant [9]. Beard et al. suggested that achieving a MIC of at least 5 points on the OKS from baseline is necessary for a clinical improvement to be deemed consequential [10].

The objective of this study was to compare PROMs for first- and second-side surgeries in patients undergoing staged BTKRs. The analysis focused on PROMs improvement, MIC, body mass index (BMI), and perioperative complications to determine if outcomes from the first side can predict results on the second side. These findings could help set patient expectations for second-side surgery and should be integrated into the informed consent process as stipulated by the General Medical Council (GMC) [11].

## Materials and methods

A detailed retrospective review was conducted utilizing a prospectively updated database maintained by a single surgeon to evaluate a cohort of staged BTKRs performed between August 2009 and January 2020. This review specifically focused on assessing the outcomes of consecutive staged BTKRs conducted by the surgeon, ensuring consistency in surgical technique and patient selection.

The surgical technique employed in all cases was standardized, involving unconstrained cemented TKRs without the use of patellar resurfacing. Implants from two companies were used: the SIGMA PFC (press fit condylar) knee (SIGMA Total Knee System) designed by DePuy Synthes (Raynham, USA) and the Triathlon Total Knee System developed by Stryker (Kalamazoo, USA). The surgical approach was uniformly applied across all surgeries to minimize procedural variability. Patient outcomes were tracked through the OKS, which were recorded at three key time points: preoperatively, at six weeks, and at one year postoperatively. The MIC was established as an OKS change of 5 points or more, in accordance with the criteria set by Beard et al. [10]. This threshold was used to evaluate whether the changes in patient-reported outcomes were clinically significant.

Inclusion criteria for the study mandated that only staged BTKRs performed by the same surgeon using the same surgical approach were included. Specifically, the cases had to involve a standard cementation technique and unconstrained implants, with no patellar resurfacing performed. Furthermore, to ensure a comprehensive assessment of outcomes, only patients with a minimum follow-up period of one year were included in the study.

Patients who had undergone unilateral TKR or revision TKR were excluded from the analysis. This exclusion criterion was set to focus on the specific cohort of bilateral staged surgeries and avoid potential confounding factors introduced by different surgical types.

Data extraction was conducted through a review of prospectively collected surgical documentation. This included detailed records of perioperative complications, as well as long-term follow-up information. To ensure accuracy and completeness, the data from the prospectively updated database was cross-verified with patient and operative records from an online hospital system. Ethical approval was not required for this study, as it involved a review of routinely collected data. However, formal approval was obtained from the health board’s research and development department to proceed with the study. Patient data was anonymized to maintain confidentiality and comply with ethical standards.

The primary outcome of the study was to assess the mean improvement in OKS between the first- and second-side surgeries in staged BTKRs. Additionally, the MIC was evaluated for each side to determine the clinical significance of the improvements. The study also analyzed patient demographics to identify factors that might influence overall outcomes. Examples include patient age at the time of first-side surgery, laterality of first-side surgery, BMI, sex and American Society of Anesthesiologists (ASA) grade.

Secondary outcomes included a detailed examination of differences in implant sizes between the first and second sides within the same patient. The study further compared patients who did not achieve MIC on one side with those who achieved MIC on the other side. Additionally, the mean improvement and MIC differences in PROMs were analyzed by comparing the outcomes of the second side TKR with a concurrent cohort of unilateral TKRs performed by the same surgeon using the same technique during the same period.

Statistical analysis of the anonymized data set was performed using Statistical Package for the Social Sciences (IBM SPSS Statistics for Windows, IBM Corp., Version 27.0, Armonk, NY). Continuous variables were evaluated using paired t-tests for within-group comparisons and independent sample t-tests for between-group comparisons. Categorical variables were analyzed using Chi-squared tests to identify significant differences in proportions. These statistical methods were employed to ensure robust analysis and to determine the significance of the observed outcomes.

## Results

**Figure 1:**
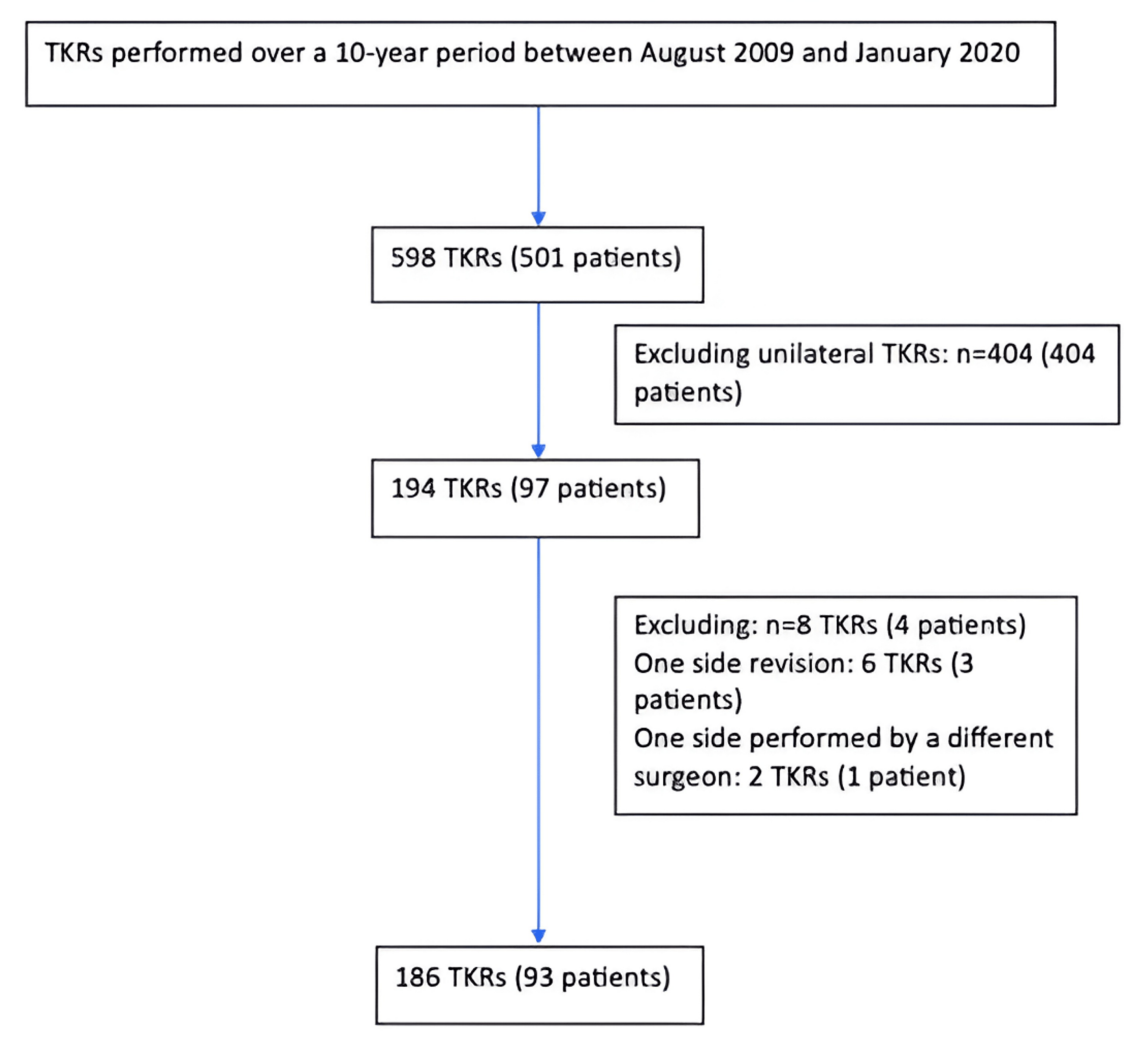
All total knee replacements (TKRs) conducted by the same surgeon during the specified study period were included. The criteria for selecting the study group and the reasons for excluding certain cases were as shown.

A total of 93 consecutive patients undergoing non-simultaneous staged BTKRs were included in the study, comprising 186 joints. This cohort was selected from an initial group of 97 bilateral procedures (194 joints) and 404 unilateral elective primary procedures performed during the same study period. The reasons for excluding four patients with bilateral TKRs are detailed in Figure 1. All patients were operated on for osteoarthritis (OA). Patient demographics and implant details are presented in Table 1.

**Table 1:** Patients’ demographics and implant type ASA: American Society of Anesthesiologists; PFC: press fit condylar knee

Number of patients (%) (n=93)		
Sex	Male	39 (42%)
	Female	54 (58%)
Age at the time of first side	40-60	23 (24.7%)
	60-80	63 (67.7%)
	>80	7 (7.5%)
First side laterality	Right	53 (57%)
	Left	40 (43%)
BMI	<25	5 (5.37%)
	25-30	13 (13.97%)
	30-40	45 (48.38%)
	>40	20 (21.5%)
	N/A	10 (10.86%)
ASA grade	1	13 (13.97)
	2	27 (29.03%)
	3	45 (48.38)
	4	5 (5.37%)
	N/A	3 (3.22%)
Implant type (n=186)		
Company	SIGMA PFC	173 (93.01%)
	Triathlon	13 (6.99%)
Cruciate retaining/substituting	Cruciate retaining	166 (78.5%)
	Cruciate substituting	20 (21.5%)
Femoral size	2 to 3	99 (53.2%)
	4 to 6	84 (45.1%)
	N/A	3 (1.6%)
Tibial size	2 to 3	103 (55.4%)
	4 to 7	80 (43.01%)
	N/A	3 (1.6%)

The average age of patients undergoing first-side surgery was 65.3 years (range: 45-88), and for second-side surgery, it was 67.7 years (range: 47-88) (Table 2). Most patients were aged 60 years or older (n=70/93, 75.2%) and 58.1% (n=54/93) were female. At the time of the first surgery, 69.8% of patients (n=65/93) had a BMI greater than 30. The mean BMI increased from 35.1 kg/m² (range: 19-52) for the first side to 35.9 kg/m² (range: 18-55) for the second side (Table 2). The mean interval between the first- and second-side surgeries was 28 months (range: 3-100 months) (Table 3).

**Table 2:** Mean BMI and age changes for first- and second-side total knee replacements

	At the time of first side	At the time of second side
BMI	Mean: 35.096, range: 19-52	Mean: 35.837, range: 18-55; p = 0.002
Age (years)	Mean: 65.32, range: 45-88	Mean: 67.74, range: 47-88; p = 0.07

**Table 3:** Time interval between staged total knee replacements

Interval between the first and second side (months)	n=93
<6	2 (2.15%)
6-12	21 (22.6%)
>12	70 (75.2%)

The primary SIGMA PFC implant was used in 93% of TKRs (173/186). Thirteen procedures out of the 186 utilised the Triathlon (Stryker Orthopaedics) implant. Nine of the 93 patients (9.67%) had differing implant systems used between the first- and second-side surgeries. All implants were cemented. The majority received cruciate retaining implants with posterior stabilised implants reserved for 8/93 (8.60%) patients with a valgus alignment.

Regarding implant size differences, 84/93 patients were analysed. Nine were excluded as these patients had differing implant systems between the first and second side. For the femoral component, the size difference was 0 ± 1 in 82/84 patients (97.6%) and 0 ± 2 in 2/84 patients (2.38%). For the tibial component, the size difference was 0 ± 1 in 81/84 patients (96.4%) and 0 ± 2 in 3/84 patients (3.57%). For the polyethylene component, the size difference was ±2 in 70/84 patients (83.3%) and ±3 in 14/84 patients (16.7%).

Follow-up

One-year follow-up PROMs were available for 97.8% (n=91/93) of patients after the first-side surgery and 89.2% (n=83/93) after the second-side surgery. At one year, the average improvement in OKS was 18.3 for the first side, with 95.6% (87/91) achieving the MIC. For the second side, the mean OKS improvement was 15.8, and 87.9% (73/83) reached MIC (p=0.340) (Table 4).

**Table 4:** Mean follow-up PROMs for first- and second-side surgery at one year MIC: minimal important change; PROM: patient-reported outcome measure

	No. of responses (%) n=93	Mean change in PROMs at one year (preoperative to one year)	MIC achieved	MIC not achieved
First side	91 (97.8%)	18.3 (range: 6 to 40)	95.6% (n = 87/91)	5.4% (n = 4)
Second side	83 (89.2%)	15.8 (range: -5 to 35) (p = 0.108)	87.9% (n = 73/83) (p = 0.340)	12.04% (n = 10) (p = 0.068)

A total of 14 out of 186 TKRs performed (7.52%) failed to achieve MIC at one-year follow-up. These 14 operations were performed on 13 patients, consisting of eight males and five females. Out of these 13 patients, three (23.1%) versus nine (61.5%) failed to reach MIC at one year following first- and second-side surgery, respectively. Only one out of 13 failed to reach MIC at one year for both first- and second-side surgeries. Out of the 14 TKRs that failed to reach MIC at one year, 10 did, however, go on to achieve MIC at two years postoperatively, with a mean OKS improvement of 24.9 (range: 9-43). No intraoperative complications were identified in all 93 patients included in the study (Table 5).

**Table 5:** Complications for both sides MIC: minimal important change; DVT: deep vein thrombosis

	Intraoperative (n = 93)	One year postoperatively (n = 93)	MIC not achieved	Early postoperative death (within 90 days)
First side	None	n = 2 (2.15%); DVT (n = 1, 1.08%); dislocation (n = 1, 1.08%)	None	N/A
Second side	None	n = 1, 1.08%; myocardial infarctions (n = 1, 1.08%)	N/A (no follow-up available)	Necrotic bowel (n = 1, 1.08%)

Analysis of the 12 patients who failed to achieve MIC for a single side found the average time interval between first- and second-side surgery was 29 months (range: 6-76 months). Preoperative BMI was 37.4 for the side that achieved MIC. This increased to 38.7 for the side that did not achieve MIC, although this was not statistically significant (p=0.29). Regarding differences in implant sizes, 9/12 patients had femoral components that were of the same size bilaterally. Three out of 12 had smaller femoral components inserted into the side that did not achieve MIC. Similar trends were identified when analysing tibial components. Nine out of 12 patients had tibial components that were of the same size bilaterally. Three out of 12 had smaller tibial components inserted into the side that did not achieve MIC (Table 6).

**Table 6:** Differences in patients not achieving MIC for a single side MIC: minimal important change; PFC: press fit condylar knee

Variable	Side achieved MIC (n=12)	Side not achieved MIC (n=12)
Age	67.6 (SD 11.41)	68.2 (SD 11.04) p = 0.72
BMI	37.4 (SD 7.23)	38.7 (SD 7.07) p = 0.29
Laterality		
Right	5 (41.67%)	-
Left	7 (58.33%) p = 0.34	-
Implant brand		
SIGMA PFC	10	12
Triathalon	2	0
Cruciate condition		
Retaining	12	12
Substituting	0	0
Femoral implant size		
Equal	9 (75%)	-
Larger	3 (25%)	-
Smaller	0	-
Tibial implant size		
Equal	9 (75%)	-
Larger	3 (25%)	-
Smaller	0	-
Polyethylene size		
Equal	2	-
Larger	5	-
Smaller	5	-

One patient did not achieve MIC for both sides, at a one-year follow-up. This patient had a time interval of 11 months between first- and second-side operations. The patient’s BMI was 25 for the first side operated on. This increased to 29 for the second side. Both sides utilised the SIGMA PFC cruciate retaining implant. The femoral components were within one size of the other and the tibial components were of the same size. Both the first- and second-side operated went on to achieve MIC at two-year follow-ups.

Ten out of the 14 TKRs that did not achieve MIC at one year postoperatively for a particular side went on to achieve MIC at two years postoperatively. Analysis of the remaining four TKRs that did not achieve MIC at two years revealed three were performed in females. Two out of the four TKRs failed to reach MIC at two years following first-side surgery and two following second-side surgery. The mean BMI amongst the patients was 43 kg/m^2^ (range: 34-52 kg/m^2^) - markedly higher in this group compared to the whole cohort. The mean time interval between first- and second-side surgery was 52 months; three out of the four had an interval of >56 months. The mean age amongst this cohort for the side not achieving MIC was 64 years (range: 56-73 years).

On an individual basis, one patient out of the group of four had a BMI of 52 and the use of a tourniquet was not feasible during the TKR. One patient suffered from a fall at six months postoperatively, yet no fracture was sustained. Although this patient did not achieve MIC at two years, they were overall satisfied with the outcome. The third patient had known spinal canal stenosis and a few years postoperatively was discovered to have a low mood requiring medical management. For the fourth patient, no review was available between two and five years postoperatively. This patient returned to the clinic for postoperative review following second-side surgery and reported a positive surgical outcome following first-side surgery.

**Figure 2:**
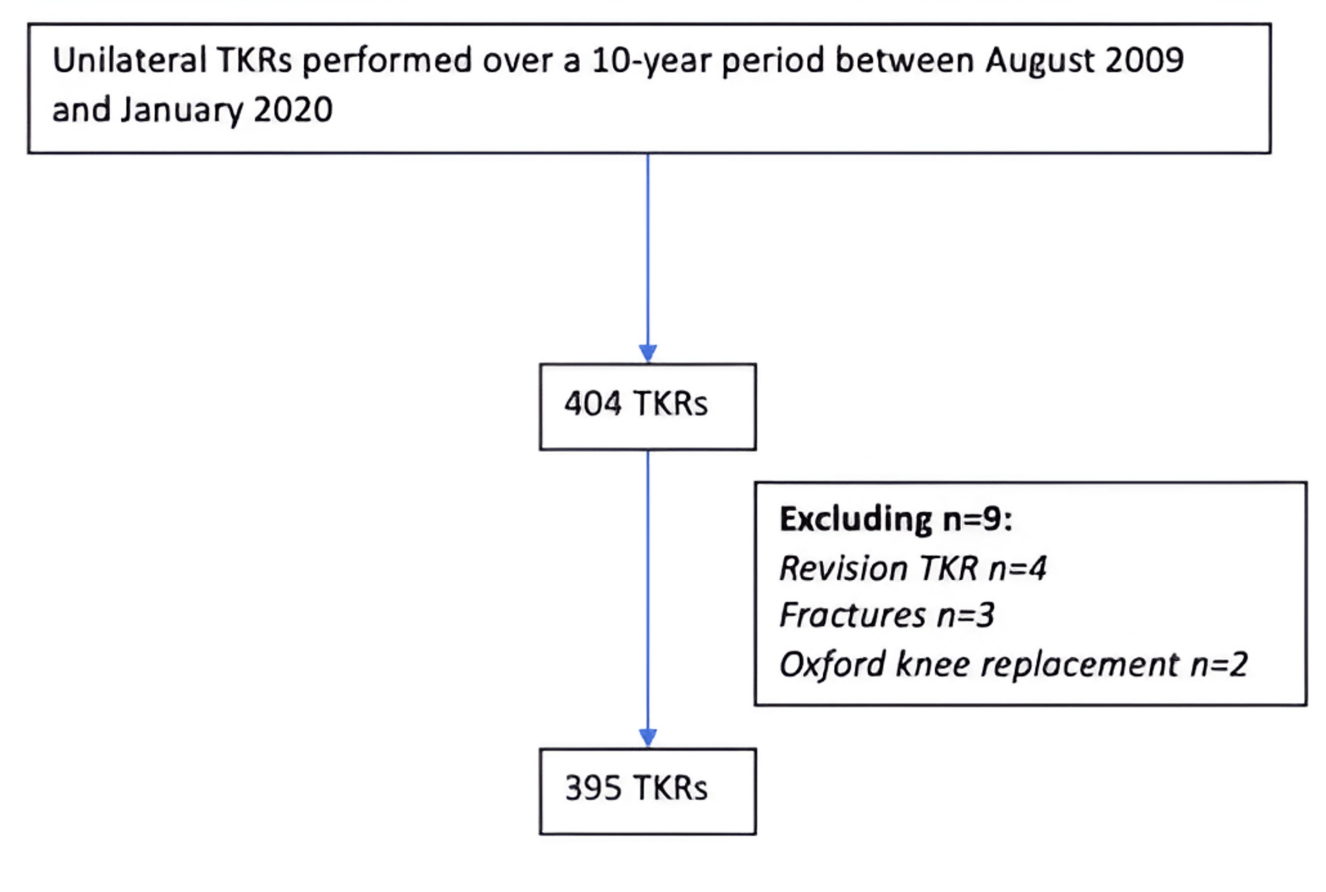
Unilateral total knee replacements (TKRs) included in secondary outcome analysis

In analyzing secondary outcomes, we compared the PROMs for the second-side surgeries with those from unilateral TKRs performed concurrently by the same surgical team, following the exclusion criteria outlined in the methodology. A total of 395 unilateral TKRs were identified and deemed eligible for analysis (Figure 2). The mean change in PROMs at one year for unilateral TKRs was 14.3, while for the second-side staged bilateral TKRs, the mean change was 15.8. The proportion of patients achieving the MIC was 81.8% in the unilateral TKR group compared to 87.9% for the second-side surgery in the bilateral staged group (p=0.89) (Table 7).

**Table 7:** Mean follow-up PROMs at second side in staged bilateral and unilateral TKRs at one year TKR: total knee replacement; PROMs: patient-reported outcome measures; MIC: minimal important change

TKR	Total number of responses at one year	Mean change in PROMs (preoperatively to one year)	MIC achieved at one year	MIC not achieved at one year
Unilateral	77.7% (n = 307/395)	14.34	81.76% (n = 251/307)	18.24% (n = 56/307)
Second side staged bilateral	89.2% (n = 83/93)	15.83 (p = 0.069)	87.95% (n = 73/83) (p = 0.89)	12.05% (n = 10/83) (p = 0.21)

## Discussion

This study found no significant difference in OKS improvements between first- (18.3) and second-side (15.8) surgery in staged BTKRs at one-year follow-up (p=0.503). Additionally, the failure to reach MIC on one side does not predict the failure of the contralateral side.

A total of 14 out of 186 TKRs performed (7.52%) failed to achieve MIC at one-year follow-up. These 14 operations were performed in 13 patients. Only one patient out of the 13 did not achieve MIC for both sides at one-year follow-up. However, at two years postoperatively, analysis revealed this patient achieved MIC bilaterally. The time interval between first- and second-side surgery was 11 months in this patient. Consequently, the failure to reach MIC in one year could be explained by the fact there was an overlap in OKS for the first- and second-side operations. Once there was an improvement in the second-side OKS, there was a subsequent improvement in the OKS for the first side operated on.

The National Joint Registry (NJR) 2023 provides one-year follow-up and OKS availability for only 26,838 out of 74,082 TKRs (36.2%) performed between April 2021 and March 2022 [7]. Our study had excellent one-year follow-up rates of 97.8% and 89.2% for first- and second-side surgery, respectively. The NJR 2023 data revealed that 95% of respondents reported an improvement following a TKR with an average health gain on the OKS being 17.3 [7]. However, the NJR results did not take into consideration the MIC, i.e., an improvement beyond error that is clinically significant [9].

Regarding satisfaction scores, the NJR 2023 found only 87% described the results of their TKR as good, very good or excellent [7]. Regarding success scores, 75.6% reported feeling much better and 14.7% reported feeling a little better following their operation [7]. Conversely, our results revealed that in one year, the mean improvement in OKS was 18.3, with 95.6% (87/91) achieving a MIC following first-side surgery. Following second-side surgery, the mean OKS improvement was 15.8, with 87.9% (73/83) achieving MIC (p=0.340). The results, however, were not statistically significant from the first side.

Regarding the 14 TKRs that did not initially achieve MIC at one year, our study revealed a high chance of improvement at two years postoperatively, with 10/14 reaching MIC by this stage (71.4%). Three out of the four patients who failed to achieve MIC at two years were females. Our results are consistent with those described by Tucker et al., who retrospectively evaluated staged bilateral primary TKRs performed by the same surgeon, using the same implant and technique [12]. They included 114 TKRs in their study. They concluded female patients undergoing second-side TKR showed less improvement in Oxford and pain scores compared to the first side [12]. The difference in outcome following second-side surgery did not apply to male patients undergoing a TKR [12].

Another significant correlation that was identified in this study was that of a higher BMI and increased likelihood of failure to achieve MIC at one year. Analysis of the 12 patients who failed to achieve MIC for a single side found preoperative BMI was 37.4 for the side that achieved MIC. This increased to 38.7 for the side that did not achieve MIC. Pozzobon et al. performed a systematic review and meta-analysis to identify whether obesity is a predictor of clinical outcomes in patients undergoing elective knee arthroplasty due to OA [13]. Sixty-two papers were included in the systematic review, of which 31 were included in the meta-analyses. They found that pre-surgical obesity is associated with worse clinical outcomes of knee arthroplasty in terms of pain, disability and complications in patients with OA [13]. Furthermore, Polat et al. aimed to evaluate the effect of morbid obesity (BMI ≥35 kg/m^2^) on functional outcomes and complication rates following uni-compartmental knee arthroplasty (UKA) [14]. One hundred and four patients were included in the analysis with an average BMI of 34.4 (range: 22-56.9). They concluded morbid obesity is an independent risk factor for functional outcomes and implant survival after UKA [14]. In addition, morbidly obese patients should be preoperatively informed about poor functional outcomes and high complication rates [14]. Therefore, treating morbid obesity before UKA should be considered [14].

Our results also demonstrated that the time interval between first- and second-side operations may be a factor in determining whether an improved outcome is achieved. The average interval between surgeries was 28 months, ranging from three to 100 months. Several factors contributed to the delay between the first- and second-side surgery in our cohort. First, co-morbid patients, including those with heart disease, diabetes, or obesity had to be managed and optimised prior to second-side surgery. Second, weight loss was a major factor, as patients were encouraged to lose weight beforehand to reduce peri-operative risks. Lastly, some patients preferred to continue managing their symptoms with non-surgical treatments, such as anti-inflammatory medications, steroid injections, and orthotics for as long as possible before deciding on surgery.

The mean time interval between first- and second-side surgery in the four patients who did not achieve MIC at two years was 52 months; an average of two years greater when compared to the whole cohort. The results suggest an earlier second-side surgery may improve satisfaction rates and postoperative outcomes. This is a common phenomenon, whereby patients who report positive outcomes following first-side surgery do not wish to wait for an inevitable deterioration before electing to have their second side operated on. In addition, patients are often keen to know whether the second knee will be functionally as good as the first [15]. Sliva et al. reported that BTKRs could be performed safely and practically four to seven days apart in a single hospitalisation [16]. Patients are, however, recommended to wait a minimum of a few months to gain maximum function from the initial procedure [15]. Other variables, such as a patient’s age, implant size, and laterality of first-side surgery, were not found to be statistically significant in our study and independent of whether MIC was achieved.

One patient did not achieve MIC for both sides, at a one-year follow-up. This patient had a preoperative OKS of 34. This improved to 38 in one year and to 41 in two years. The failure to reach MIC in one year could be explained by a ceiling effect. However, the OKS and ceiling or floor effects in knee arthroplasty patients are a highly debated topic, with multiple confounding studies. Harris et al. examined whether the OKS demonstrated a floor or a ceiling effect when used to measure the outcome of knee replacement surgery in a large national cohort of 72,154 patients [17]. Based on NHS PROMs data, the OKS was found not to exhibit a ceiling or floor effect for both its pain and functional subscales [17]. On the other hand, Clement et al. performed a retrospective analysis of 5,857 patients undergoing a primary TKA [18]. They demonstrated that a preoperative OKS of 23 or more was predictive of achieving a postoperative ceiling OKS at one year when defined as a maximal score [18].

Limitations of the study

First, this study is a retrospective analysis of a consecutive cohort of staged BTKRs performed at a single centre by a single surgeon. Our aim, however, was to reduce confounding factors that may result in bias between first- and second-side surgeries. Second, we did not review the degree or severity of preoperative OA. This may have resulted in bias, whereby patients with a poorer preoperative state demonstrate a heightened improvement in postoperative outcomes. Finally, our study evaluated only OKS. There was no comment on pain or a patient’s mental and emotional health effect on the outcome.

## Conclusions

This study found no significant difference in PROMs improvements between the first and second sides of staged BTKRs at one-year follow-up. A failure to achieve the MIC on one side does not preclude success on the other. Most patients who do not reach MIC in one year can still achieve it in two years. Persistently poor scores were linked to high BMI and female sex.
